# Acute pulmonary embolism with inferior vena caval thrombus following radiofrequency ablation of the great saphenous vein despite early ultrasound surveillance

**DOI:** 10.1016/j.jvscit.2025.101825

**Published:** 2025-04-25

**Authors:** Mitali Doshi, Juan Carlos Jimenez

**Affiliations:** Gonda Venous Center, Division of Vascular and Endovascular Surgery, David Geffen School of Medicine at UCLA, Los Angeles, CA

**Keywords:** Pulmonary embolism, Radiofrequency, Saphenous, Reflux, Varicose vein

## Abstract

The reported incidence of pulmonary embolism in the published literature after radiofrequency ablation of the great saphenous vein is exceedingly rare. Recent societal clinical practice guidelines recommend against routine postprocedural ultrasound screening for ablation-related thrombus extension in asymptomatic average-risk patients. However, screening is recommended for asymptomatic high-risk patients. We present the case of a 69-year-old woman with multiple risk factors for the development of venous thromboembolism who developed bilateral pulmonary emboli despite early postprocedural ultrasound screening. As highlighted in this paper, surveillance ultrasound cannot be solely relied upon to detect and prevent pulmonary embolus after great saphenous vein radiofrequency ablation.

The reported incidence of thromboembolic complications following thermal ablation of the great saphenous vein (GSV) is low. The combined incidence of ablation-related thrombus extension (ARTE), deep venous thrombosis (DVT), and pulmonary embolism (PE) after this treatment is estimated to be between 1% and 2%.^1^^,^^2^ The incidence of isolated PE is estimated to be exceedingly rare at 0.1%.^2^ The most recent Society for Vascular Surgery, American Venous Forum, and American Vein and Lymphatic Society guidelines recommend against routine early postprocedural duplex ultrasound surveillance in average-risk, asymptomatic patients.^3^ However, ultrasound screening of asymptomatic high-risk patients is recommended and meant to prevent and detect thrombotic complications after saphenous vein radiofrequency ablation (RFA).^3^ We present the case of a high-risk patient who developed clinically significant bilateral pulmonary emboli after RFA of the GSV despite early postprocedural ultrasound screening as advocated by societal guidelines and discuss lessons learned to possibly prevent future similar events. Patient consent was obtained to publish case details and images.

## Case report

A 69-year-old woman presented with symptomatic right leg varicose veins. Her symptoms included pain, aching, heaviness, and edema (Clinical Etiological Anatomical Physiological clinical class 3). Her symptoms were present for many years, and she was compliant with compression stockings for the past year. Her symptoms interfered with prolonged standing, exercise, and other activities of daily living.

Her medical history was significant for invasive ductal carcinoma of the right breast (stage III, T1a, N0, estrogen receptor positive) treated with lumpectomy and radiation 7 years prior. She was monitored yearly and cancer-free since her lumpectomy. She had no prior history of phlebitis or DVT. Her family history was negative for any known hypercoagulable disorders. She had a 30 pack-year smoking history, but quit 20 years prior. Medications included an etonogestrel/ethinyl estradiol-secreting vaginal ring (0.120 mg/0.015 mg/day) changed monthly, prescribed by per primary care physician for incontinence and vaginal dryness.^4^ She also took levothyroxine (Synthroid) 75 μg/day. Her body mass index was 31 kg/m^2^. On physical examination, she had superficial varicosities, most prominent on the right medial thigh, and right ankle edema.

**Fig 1:**
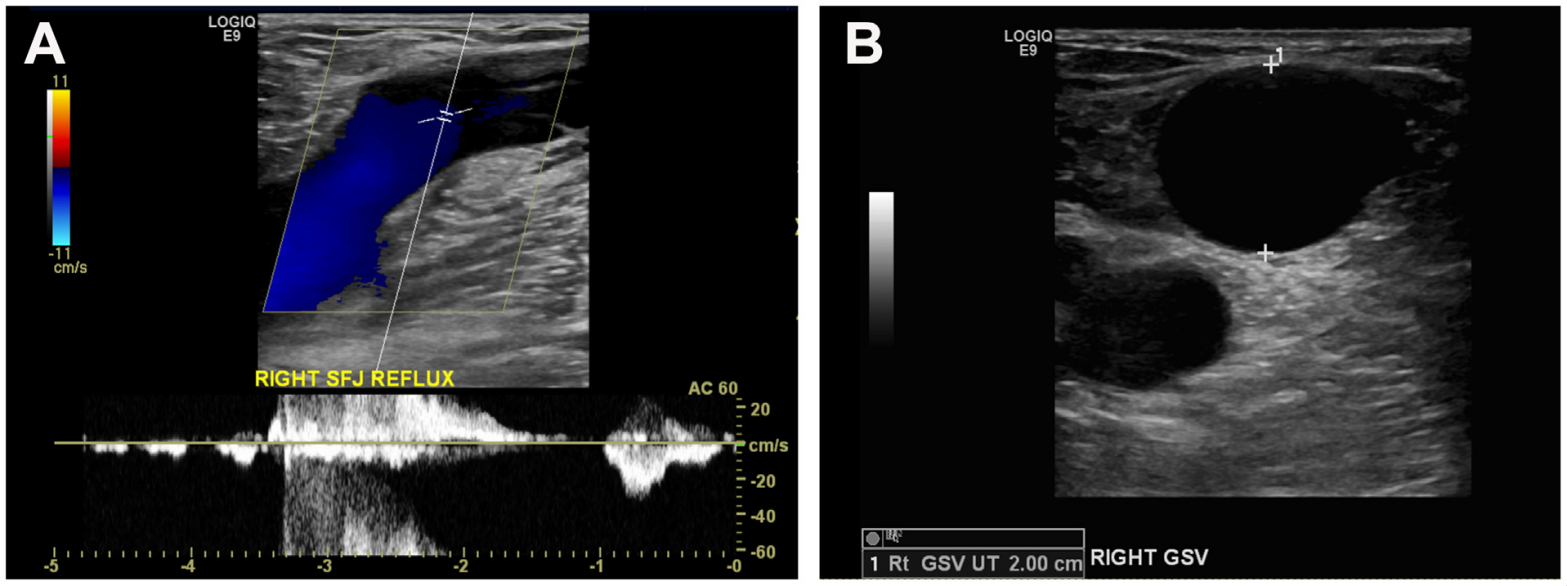
**(A)** Preprocedural duplex ultrasound examination demonstrating reflux at the right saphenofemoral junction (*SFJ*). **(B)** Preprocedural duplex ultrasound examination demonstrating a 20-mm great saphenous vein (*GSV*) caudal to the right SFL.

A preprocedural duplex ultrasound demonstrated an incompetent right saphenofemoral junction (SFJ) with 3 seconds of reflux (Fig 1, *A*). Her GSV diameter measured 20 mm immediately caudal to the SFJ and upper thigh and 10 mm in the mid-thigh (Fig 1, *B*). A 5-mm anterior superficial tributary vein branched in the distal thigh and became anatomically superficial. Caudal to this point, the GSV became small (3 mm) to the level of the distal calf. Right common femoral vein (CFV) reflux (>0.5 seconds) was also present.

RFA of the right GSV was performed in the ambulatory procedure unit under local anesthesia. Ultrasound-guided percutaneous access was obtained in the distal thigh, where the GSV branched with a micropuncture needle and wire. A 7F sheath was inserted into the GSV over the wire. A 7F ClosureFast RFA catheter (Medtronic, Minneapolis, MN) with a 7-cm treatment length was advanced into the GSV, and its tip was secured 2.5 cm caudal to the SFJ. A spinal needle was used to inject a tumescent solution (normal saline, 500 mL: 1% lidocaine 50 mL: sodium bicarbonate 5 mL) into the perivenous tissue surrounding the GSV. Approximately 100 mL of total tumescent solution was used. The distance of the catheter from the SFJ was confirmed with ultrasound before and after the tumescence injection and collapse of the vein around the catheter was confirmed prior to RFA. Two 20-second treatments at 120° Celsius were performed in the GSV segment 2.5 cm caudal to the SFV. The catheter was then withdrawn 7 cm, and two additional 20-second treatments were performed at this level. The sheath and catheter were removed, and the leg was compressed. She tolerated the procedure without difficulty. The duration of the procedure was 35 minutes. Per patient wish, no concomitant procedures (ie, phlebectomy, sclerotherapy) were performed.

**Fig 2:**
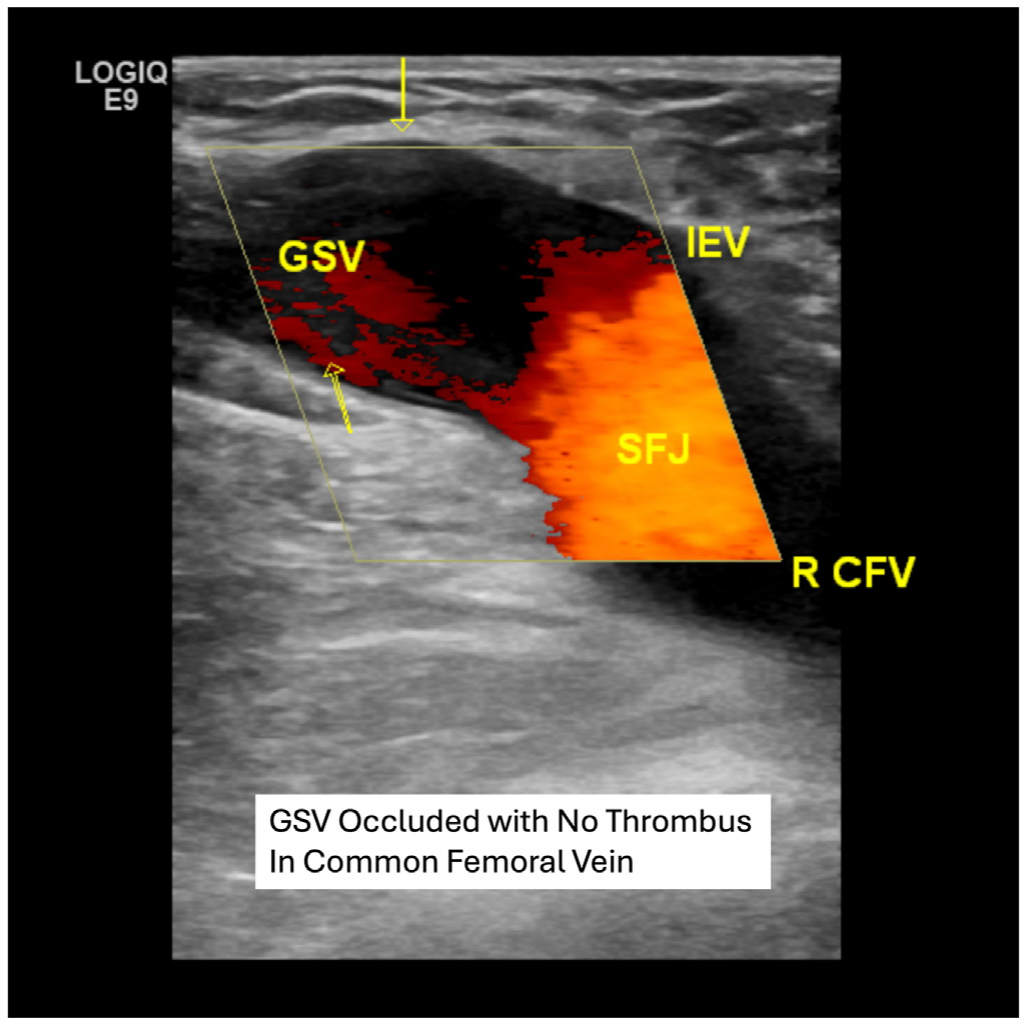
Initial postprocedural duplex examination 2 days after radiofrequency ablation (RFA) ultrasound examination demonstrating successful occlusion of the great saphenous vein with no ablation-related thrombus extension (ARTE) and a patent R CFV. *GSV*, great saphenous vein; *IEV*, inferior epigastric vein; *R CFV*, right common femoral vein; *SFJ*, saphenofemoral junction.

The patient returned 2 days later for a routine postprocedure duplex ultrasound examination to rule out ARTE and ensure GSV closure. She was asymptomatic. The ultrasound performed by an ultrasound technician in our accredited vascular laboratory demonstrated successful closure of the right GSV from the SFJ to the distal thigh and a patent CFV with no evidence of ARTE or DVT (Fig 2). A complete ultrasound study was performed and demonstrated no evidence of infrainguinal DVT from the groin to the ankle. The patient was instructed to ambulate as tolerated, and the patient was wearing postprocedure compression with compliance per the guidelines of the Society for Vascular Surgery and the American Venous Forum.

**Fig 3:**
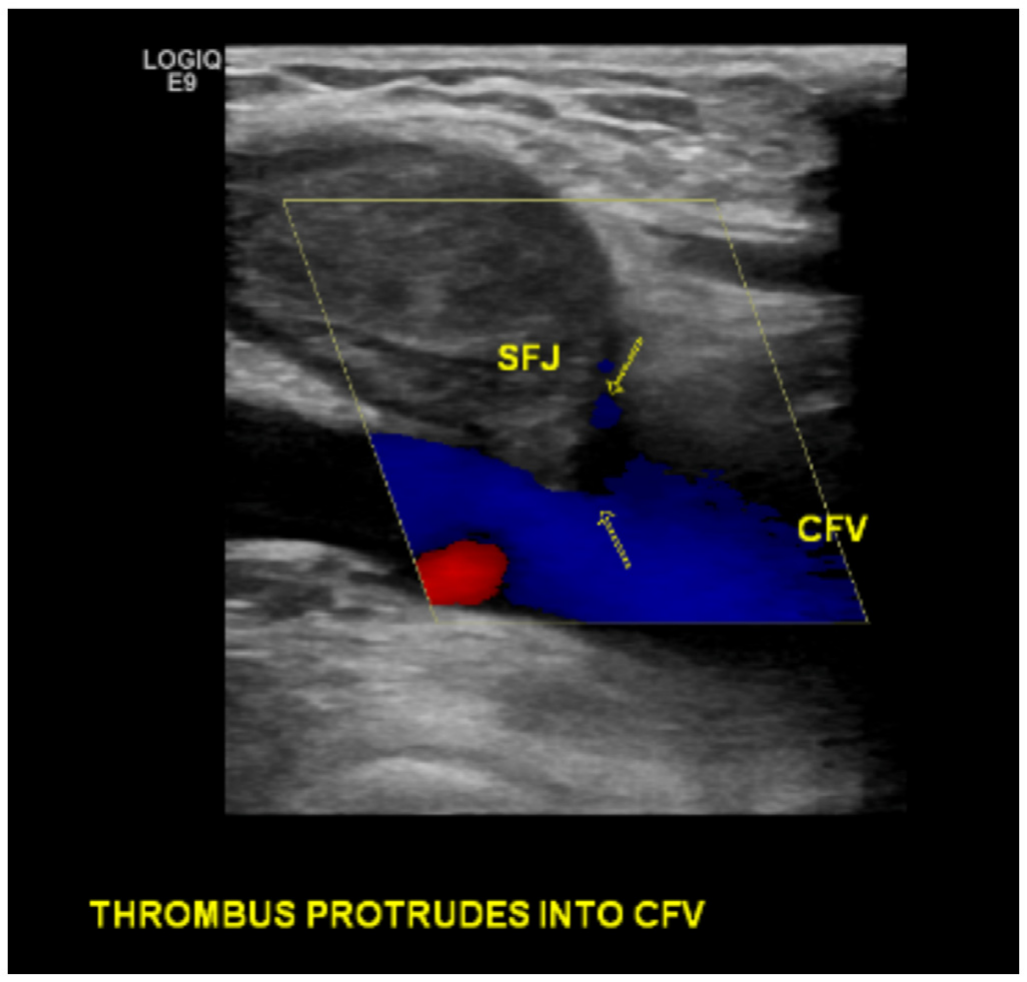
Postprocedural duplex ultrasound examination 7 days after radiofrequency ablation (RFA) demonstrating ablation-related thrombus extension (ARTE). The patient presented with worsening shortness of breath and cough. *CFV*, common femoral vein; *SFJ*, saphenofemoral junction.

**Fig 4:**
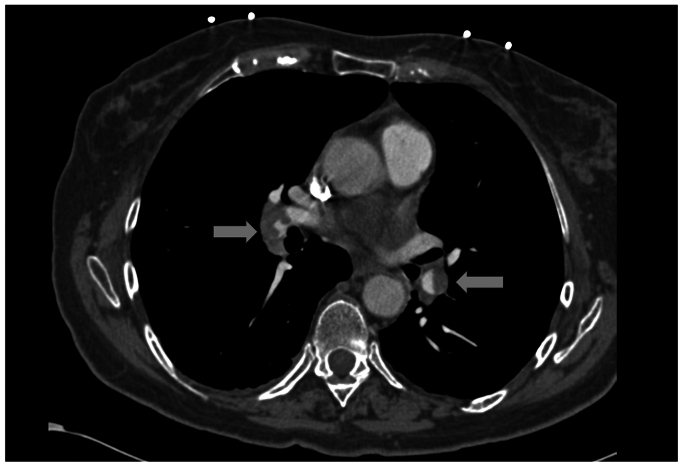
Computed tomography angiogram demonstrating bilateral pulmonary emboli.

**Fig 5:**
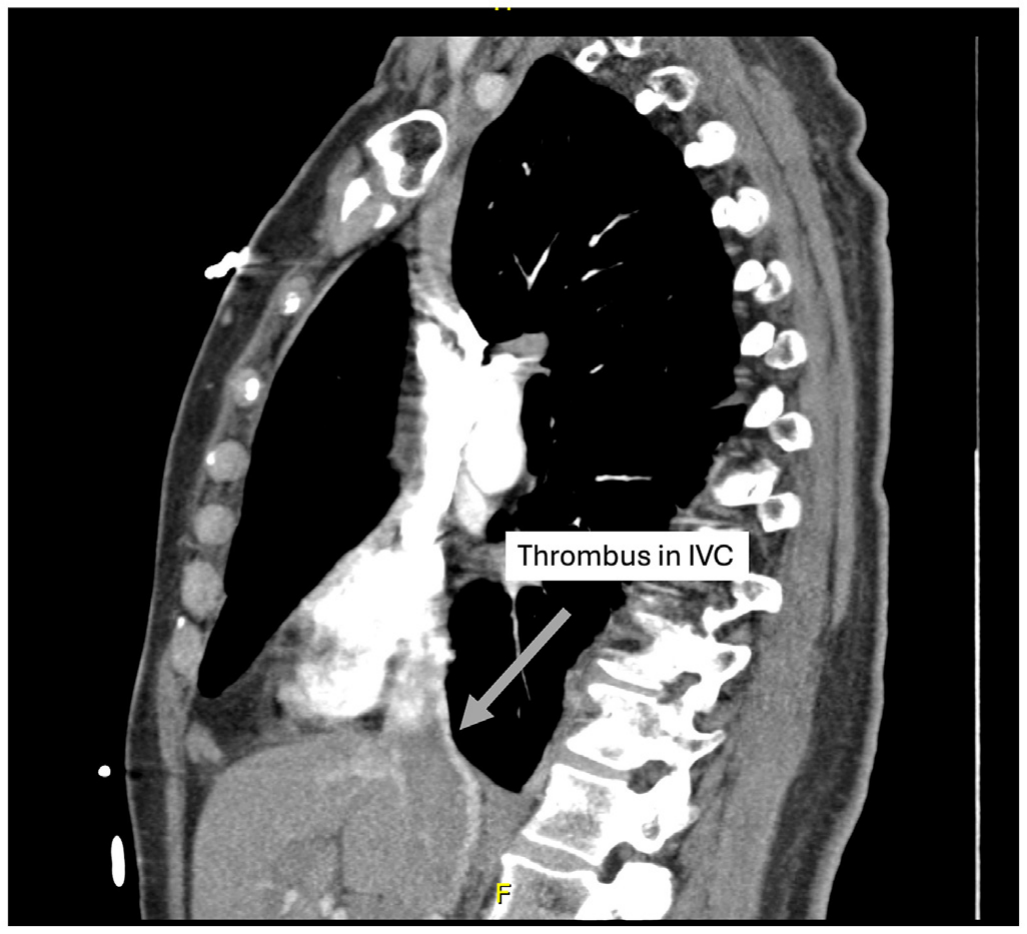
Computed tomography angiogram demonstrating thrombus in the supradiaphragmatic inferior vena cava (*IVC*).

Five days later, she presented to our clinic with shortness of breath and cough, which developed 1 day after her initial duplex ultrasound examination and was persistent for 4 days. She was tachypneic and was noted to have 98% oxygen saturation on room air. A repeat duplex ultrasound examination demonstrated ARTE in the CFV (Fig 3). She was sent immediately to the emergency room for a higher level of care. A heparin bolus (71 mg/kg) was administered, and a heparin drip was titrated to a goal partial thromboplastin time of 50 to 80 seconds. Computed tomography angiography demonstrated acute multifocal bilateral PE (Fig 4). Thrombus burden in the inferior vena cava extending into the right atrium was also visualized (Fig 5). Her hormonal therapy was discontinued. Transthoracic echocardiogram demonstrated mild right atrial enlargement with no evidence of right heart strain and a normal ejection fraction. Her tachypnea improved significantly with systemic anticoagulation, and the patient remained hemodynamically stable. Thus, thrombolysis and aspiration thrombectomy were not performed. The hematology service was consulted, and an extensive hypercoagulable workup was conducted, which was negative. Magnetic resonance venography of the chest was performed on hospital day 3, and persistent bilateral pulmonary emboli were demonstrated, but there was restoration of patency and resolution of thrombus in the IVC. The patient was discharged home on hospital day 4 and transitioned to oral coumadin bridged with enoxaparin sodium (Lovenox). At her 6-month follow-up visit, she was asymptomatic. Repeat computed tomography angiography 6 months later demonstrated resolution of her bilateral PEs. Her oral anticoagulation was discontinued at this time.

## Discussion

Although rare, life-threatening thromboembolic complications after endovenous varicose vein treatments may occur. Despite recent societal guidelines' recommendations that average-risk asymptomatic patients not be screened, they recommend that early duplex ultrasound examination be performed in high-risk asymptomatic patients undergoing thermal saphenous vein ablation. However, the current guidelines do not specify a preferred time interval for postprocedure surveillance. In this case, seems appears the duplex examination 48 hours after RFA was too early, and a duplex examination at 5 to 7 days may have been more appropriate.

This patient had several factors associated with an increased risk of thromboembolic events. Her GSV diameter was 20 mm immediately caudal to the SFJ and 10 mm to the mid-thigh level. Increased vein diameters (>8 mm) are associated with a higher risk of ARTE in recent published studies.^5^, ^6^, ^7^ Multivariate analysis from a study at our institution after 1000 consecutive saphenous vein RFAs demonstrated an increased risk of ARTE in patients with GSV diameters larger than 8 mm.^8^ In a review of 6707 saphenous veins treated with RFA by Sufian et a^9^, GSV diameters of greater than 10 mm were associated with a higher risk of ARTE, and two nonfatal PEs (0.03%) occurred in their study.

Other risk factors included age, class 1 obesity, her history of breast cancer, and use of an etonogestrel/ethinyl-estradiol secreting vaginal ring. The risk of DVT has been found to increase significantly after age 45, and being older than 60 has been cited as a strong risk factor.^10^ Although her breast cancer was treated years earlier, there is evidence suggesting persistent prothrombotic states following prior cancer diagnoses.^11^, ^12^, ^13^, ^14^ Obesity (body mass index >30) has been demonstrated to be an independent risk factor for the development of thrombotic complications after varicose vein treatments.^15^, ^16^, ^17^ The long-term risk of venous thromboembolism in breast cancer patients has been found in multiple studies.^18^, ^19^, ^20^ Higher thrombotic risks have also been reported with the use of exogenous estrogen therapy and in a study of more than 1 million women by Lidegaard et al,^21^ women who used vaginal rings had a 6.5 times risk of confirmed venous thrombosis compared with nonusers of hormonal contraception of the same age. Despite being high risk for ARTE after RFA based on her medical history and appropriately screened based on societal guidelines, the immediate postprocedure duplex did not demonstrate ARTE. It was not helpful in the prevention of her PE. Seven days later, when her follow-up duplex ultrasound examination demonstrated ARTE, she was already symptomatic with cough and shortness of breath.

Discontinuation of her exogenous hormone therapy may have decreased her postprocedure thromboembolic risk. Still, there is insufficient evidence in the peer-reviewed literature and no formal clinical guidelines addressing this topic. Some authors have suggested that periprocedural prophylaxis with low-molecular-weight heparin may decrease the risk of ARTE in patients with large diameter veins, although no consensus exists.^5^^,^^22^ Another clinical option would have been to perform high ligation and stripping (HL/S) of the GSV instead of RFA to avoid direct communication between the CFV and the thrombus in the occluded vein. However, HL/S has also been associated with postoperative DVT.^23^ However, because HL/S allows for flush ligation at the SFJ this patient may have been a better candidate for this surgical treatment compared with RFA.

When faced with a patient with large diameter saphenous veins and numerous possible risk factors for DVT, enhanced postprocedure duplex surveillance should be considered, given the possible heightened risk of ARTE and DVT. Patients should be counseled appropriately that, although rare, life-threatening risks associated with elective thermal ablation of the saphenous vein can occur. Careful identification of risk factors for ARTE and venous thromboembolism should be made with a detailed medical history. Periprocedural alteration of the patient's medication regimen before RFA (ie, prophylactic anticoagulation, stopping preprocedural exogenous hormonal therapy) should be considered along with the patient and other medical providers offering care. As this case highlights, postprocedural duplex ultrasound examination will not always be able to diagnose acute DVT and prevent PE after saphenous vein RFA.

## Funding

None.

## Disclosures

None.
